# Toward sustainable LDPE packaging films from cotton straw: barrier–moisture–microenvironment coupling for food preservation

**DOI:** 10.1016/j.fochx.2026.103917

**Published:** 2026-04-24

**Authors:** Chi-Hui Tsou, Nuo Xu, Jia Zheng, Genjun Ye, Lin-Kai Wu, Xin Huang, Tao Guo, Yulong Luo, Guangfu Mao, Xue-Fei Hu

**Affiliations:** a Material Corrosion and Protection Key Laboratory of Sichuan Province, School of Materials Science and Engineering, Sichuan University of Science and Engineering, Zigong 643000, China; b Wuliangye Yibin Co., Ltd., Yibin, Sichuan 644000, China

**Keywords:** LDPE composites, Cotton straw, Interfacial compatibilization, Moisture regulation, Gas barrier, Food packaging

## Abstract

Sustainable food packaging that preserves quality without migratory additives is highly desirable. While CS–LDPE composites have been widely studied for structural applications, their role in food packaging remains limited. Herein, cotton straw (CS) is used as a bio-filler in LDPE films via melt blending compatible with conventional processing. With LDPE-g-MA compatibilization, interfacial densification and modified surface morphology are achieved, enabling coupled regulation of gas transport, moisture behavior, and the local microenvironment. Surface roughness variation further reflects these interfacial changes and their influence on moisture interaction. At moderate CS contents (10–15 wt%), oxygen transmission is reduced by up to ∼55% while maintaining balanced water vapor permeability. These effects translate into improved preservation, including reduced weight loss, delayed deterioration, stabilized pH, and reduced microbial growth. The effect is attributed to structure-regulated mass transport rather than direct antimicrobial activity, offering a scalable strategy for LDPE-based food packaging.

## Highlights

• Cotton straw serves as a multifunctional bio-filler in LDPE packaging films.
• LDPE-g-MA improves interfacial densification and filler dispersion.
• Moderate cotton straw loadings reduce oxygen transmission.
• Water vapor permeability remains balanced, limiting excessive dehydration.
• Structural regulation delays food deterioration and microbial growth without added antimicrobial agents.

## Graphical abstract

**Figure:**
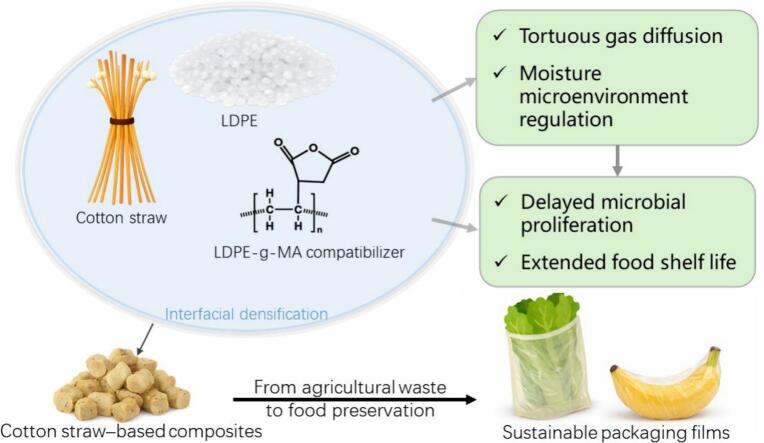

## 1 Introduction

The increasing demand for sustainable food packaging materials has stimulated extensive research into polymer-based systems capable of balancing mechanical integrity, barrier performance, and food preservation functionality. Among conventional polymers, low-density polyethylene (LDPE) remains one of the most widely used materials in food packaging due to its excellent processability, flexibility, and chemical resistance (Saha et al., 2022; Zia et al., 2019). However, LDPE is inherently non-biodegradable and exhibits limited gas barrier and antimicrobial performance, which restricts its applicability in environmentally friendly and functionally enhanced packaging systems (Alkarri et al., 2024; Youssef et al., 2023). Consequently, incorporating bio-based fillers derived from agricultural residues has emerged as an effective strategy to improve the sustainability profile and functional performance of LDPE-based materials (Pacheco et al., 2023; Pei et al., 2024). Such lignocellulosic residues—originating from straw, husks, and stalks—are abundant, renewable, and cost-effective, and have been shown to enhance mechanical strength, thermal behavior, and barrier properties when properly integrated into polymer matrices (Ballupete Nagaraju et al., 2023; Otto et al., 2022; Pei et al., 2024).

Cotton straw (CS) is a widely available agricultural byproduct rich in cellulose and lignin, making it a promising candidate for polymer reinforcement (Cai et al., 2024; Ding et al., 2025). Nevertheless, the hydrophilic nature of CS and its poor interfacial compatibility with hydrophobic polyolefins such as LDPE often lead to phase separation, reduced mechanical performance, and compromised moisture barrier properties in the resulting composites (Lee et al., 2021; Somsuk et al., 2025). Overcoming this interfacial incompatibility remains a key challenge in the development of high-performance bio-composites. Although numerous studies have explored the influence of natural fibers on the mechanical and barrier properties of LDPE-based systems (Sharma et al., 2021; Tamizhdurai et al., 2024), investigations specifically focused on CS-filled LDPE composites—particularly in thin-film packaging configurations—remain limited.

Previous studies on CS–LDPE systems have largely emphasized energy and environmental applications rather than packaging functionality. For example, Yuan et al. investigated the co-pyrolysis of CS and LDPE to produce biochar with enhanced heavy-metal adsorption capacity, reporting a maximum Pb(II) uptake of 199.82 mg/g at a CS-to-LDPE ratio of 1:1 and a pyrolysis temperature of 500 °C (Yuan et al., 2022). Similarly, Li et al. examined the *co*-liquefaction of CS and polyethylene under subcritical conditions and demonstrated improved bio-oil yield and hydrocarbon conversion efficiency, attributing these effects to polysaccharide-assisted β-scission and secondary reactions such as Diels–Alder condensation and hydrodeoxygenation (Li et al., 2024). More recently, Bekele et al. evaluated the effects of natural weathering on CS fibers used in LLDPE composites and found that degradation of cellulose and hemicellulose led to reduced mechanical performance and increased water uptake, highlighting the importance of fiber stability in bio-filler applications (Bekele et al., 2017). From a structural perspective, Khan et al. reported that CS–LDPE composites exhibited enhanced tensile and flexural strength at higher filler loadings, although impact resistance declined with increasing CS content (Khan et al., 2024), indicating their potential as cost-effective wood–plastic-type materials.

Despite these advances, the application of CS–LDPE composites in food packaging have received comparatively little attention. In particular, the multifunctional performance of CS-reinforced LDPE films—including crystallinity modulation, moisture sensitivity, gas barrier behavior, and actual food preservation performance—has not been systematically evaluated. A holistic investigation from a food-packaging perspective is therefore needed to clarify how CS incorporation influences both structural properties and preservation-related functionality.

Maleic anhydride–grafted polyethylene (LDPE-g-MA) has been widely employed as an effective compatibilizer to improve interfacial adhesion and compatibility between polar lignocellulosic fillers and nonpolar polymer matrices (Ahn et al., 2016; El-Wakil et al., 2022). Through chemical grafting and potential ester-bond formation, LDPE-g-MA can bridge the polarity mismatch, thereby enhancing mechanical integrity, dispersion uniformity, and moisture resistance in bio-composite systems (Telen et al., 2015).

Beyond structural reinforcement, there is growing interest in packaging films that provide auxiliary preservation benefits through moisture regulation and microbial response. Although cotton straw has not been traditionally regarded as an antimicrobial material, recent observations suggest that lignocellulosic components may indirectly influence microbial viability by modulating surface chemistry and local moisture availability. In addition, maleic anhydride functionalities have been occasionally reported to interact with microorganisms under certain conditions; however, such effects are generally weak and are not considered a primary antimicrobial mechanism (Ge et al., 2021). When incorporated as a compatibilizer rather than an active agent, LDPE-g-MA may therefore facilitate preservation-related effects primarily through interfacial densification and barrier enhancement rather than direct bactericidal action.

While active packaging strategies based on metallic or bioactive additives can provide strong antimicrobial effects, their application in large-scale polyolefin food packaging remains constrained by concerns regarding additive migration, regulatory compliance, recyclability, and long-term stability. For example, starch-based films incorporating Nisin and curcumin nanoemulsions have been reported to provide both antimicrobial activity and real-time freshness monitoring capabilities in refrigerated food systems (Ren et al., 2025). However, such systems rely on active additives, whereas the present work focuses on preservation through structural and transport regulation without migratory components. In this context, passive yet effective regulation of gas and moisture transport through interfacial and structural design represents a complementary and industrially pragmatic strategy for food preservation.

Given the continued dominance of polyolefin-based packaging in current food supply chains, drop-in strategies that reduce virgin polymer usage while retaining process compatibility are of particular practical relevance. Unlike previous CS–LDPE systems that have mainly focused on structural or energy-related applications, this work shifts the focus toward food packaging, where performance depends on the coupled regulation of gas transport, moisture behavior, and the microbial microenvironment.

In this study, LDPE/CS and LDPE-g-MA/CS (M-LDPE/CS) composite films were fabricated via melt blending and compression molding using processes compatible with conventional polyolefin manufacturing. The effects of CS loading and interfacial compatibilization on mechanical properties, crystallinity, barrier behavior, moisture regulation, microbial response, and food preservation performance were systematically investigated. Through this integrated evaluation, this work establishes a scalable strategy for converting agricultural waste into functional LDPE-based food packaging films and clarifies the role of cotton straw as a multifunctional bio-filler contributing to both structural reinforcement and preservation-related functionality. Rather than introducing a new tortuosity mechanism, this work focuses on a coupled regulation strategy, in which interfacial structure governs gas transport, moisture behavior, and the microbial microenvironment in food packaging systems.

## 2 Experimental

### 2.1 Materials and pretreatment of cotton straw

Low-density polyethylene (LDPE) was commercially procured and used as the polymer matrix. LDPE (grade 2426H, Mw ≈ 30,000; density 0.924 g/cm^3^; milky white) was supplied by the Maoming Branch of China Petroleum & Chemical Corporation (Maoming, China). Maleic anhydride-grafted low-density polyethylene (LDPE-g-MA) was used as a compatibilizer in selected formulations. Cotton straw (CS) was obtained from Sichuan Golden-Elephant Sincerity Chemical Co., Ltd. (Meishan 620,010, China) and used as the natural filler.

Prior to composite fabrication, the CS underwent a pretreatment process to ensure particle size uniformity (≤0.075 mm), which included the following steps:

(1) Pulverization: Raw cotton straw was manually cut into small segments and then ground into fine powder using a high-speed grinder. The powder was passed through a 200–300 mesh sieve to obtain particles with uniform size distribution.
(2) Drying: The sieved CS powder was dried in a vacuum oven in two stages: initially at 105 °C for 4 h to remove bulk moisture, followed by 85 °C for 8 h to ensure low residual moisture content and prevent agglomeration during melt processing.

### 2.2 Preparation of LDPE-g-MA

LDPE-g-MA was prepared by melt grafting in our previous work (Tsou et al., 2025). Briefly, LDPE was melt blended with maleic anhydride in the presence of a radical initiator under controlled temperature and shear conditions. After grafting, the product was purified to remove unreacted monomer, and the grafting degree was determined to be approximately 0.99%.

### 2.3 Preparation of LDPE/CS and M-LDPE/CS composites

**Fig. 1:**
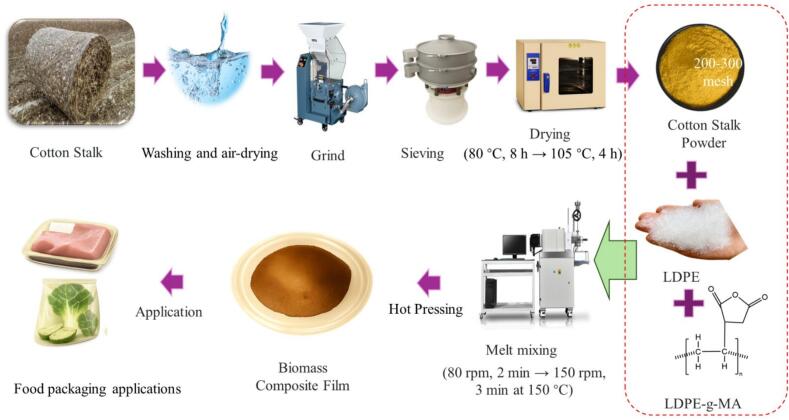
Schematic illustration of the preparation process of LDPE/CS and M-LDPE/CS composite films.

LDPE/CS and M-LDPE/CS composites were prepared by melt blending in a torque rheometer according to the formulations summarized in Table 1, and the overall preparation procedure is illustrated in Fig. 1. The preparation process involved the following steps:

(1) Final Drying of CS Powder: Before compounding, CS powder was further dried at 80 °C for 8 h and then at 105 °C for 4 h in a vacuum oven to eliminate residual moisture and avoid hydrolytic degradation or poor interfacial bonding during mixing.
(2) Weighing of Materials: LDPE, LDPE-g-MA, and CS were weighed precisely according to the formulations listed in Table 1. To prevent moisture reabsorption, weighed materials were immediately returned to the vacuum oven before use.
(3) Melt Blending: The materials were introduced into a torque rheometer in sequence. For LDPE/CS composites, LDPE and CS powders were directly fed into the chamber. For M-LDPE/CS composites, LDPE, LDPE-g-MA, and CS were blended. The mixing was conducted at 150 °C, starting with a rotor speed of 80 rpm for 2 min, followed by 150 rpm for an additional 3 min to ensure uniform dispersion of the CS filler and compatibilizer.
(4) Compression Molding: The homogenized blends were molded into thin films (∼0.3 ± 0.02 mm thickness) using a hot-press machine under a pressure of 7.5 MPa at 160 °C. After hot pressing, the films were allowed to cool to room temperature under ambient conditions. The films used for all subsequent characterization and testing were prepared with a consistent thickness of approximately 0.3 ± 0.02 mm.
(5) Specimen Preparation: The films were cut into standard dumbbell-shaped specimens using a precision cutter, suitable for mechanical testing and other characterization experiments.

**Table 1:** Formulations of LDPE, LDPE/CS, and LDPE-g-MA/CS (M-LDPE/CS) composite films. LDPE-g-MA was used as a compatibilizer at 4.5 wt% of the polymer matrix.

Samples	LDPE (wt%)	LDPE-g-MA (wt%)	Cotton straw (wt%)
LDPE	100	0	0
LDPE/CS_10	90	0	10
LDPE/CS_15	85	0	15
LDPE/CS_20	80	0	20
LDPE/CS_25	75	0	25
M-LDPE	95.5	4.5	0
M-LDPE/CS_10	85.95	4.05	10
M-LDPE/CS_15	81.175	3.825	15
M-LDPE/CS_20	76.4	3.6	20
M-LDPE/CS_25	71.625	3.375	25

### 2.4 Tensile properties

The tensile properties of the LDPE/CS and M-LDPE/CS composites were measured using a microcomputer-controlled electronic universal testing machine (model FBS10KNW). The tests were conducted according to ASTM D882-12. Specimens with dimensions of 50 mm × 6.5 mm × 1 mm were used. The testing speed was set at 10 mm/min. Each sample was tested at least five times, and the results are reported as mean ± standard deviation.

### 2.5 Fourier transform infrared spectroscopy (FTIR)

Instrumentation. FTIR spectrometer with diamond ATR accessory (Nicolet 6700, Thermo Fisher Scientific, Waltham, MA, USA). Procedure. Rectangular film pieces (∼10 × 10 mm, ∼0.3 ± 0.02 mm thick) were measured at ambient temperature in ATR mode using a constant-pressure clamp. Spectral range: 4000–600 cm^−1^; resolution: 4 cm^−1^; 32 scans per spectrum; fresh background prior to each sample. Spectra were baseline-corrected and vector-normalized. Peak positions were read from absorbance spectra with second-derivative assistance (carbonyl region 1850–1650 cm^−1^; C—O region 1250–1000 cm^−1^). All plots used identical vertical scales to compare band shifts among LDPE, M-LDPE, and M-LDPE/CS.

### 2.6 Contact angle measurement

The surface hydrophilicity of the LDPE/CS and M-LDPE/CS composites was evaluated using a contact angle goniometer (model JC2000D, Shanghai Zhongchen Digital Technology Equipment Co., Ltd., China). The contact angle measures the degree of wetting between a liquid and a solid surface. A contact angle less than 90° indicates a hydrophilic surface, where smaller angles correspond to better wettability. Conversely, a contact angle greater than 90° denotes a hydrophobic surface, with larger angles indicating poorer wettability.

For each measurement, a 2 μL droplet of distilled water was carefully deposited onto the film surface using a micro-syringe. The contact angle was recorded using the five-point fitting method at 0 s, 3 s, 6 s, 9 s, and 12 s. Each sample was measured five times, and the average value was reported. The test parameters were as follows: sample thickness, 0.3 ± 0.02 mm; ambient temperature; and repeated five measurements per sample to ensure statistical accuracy.

### 2.7 Scanning electron microscopy (SEM)

The fracture morphology of the composites after tensile testing was observed using a scanning electron microscope (SEM, model VEGA3SBU, TESCAN, Czech Republic). Prior to imaging, the samples were sputter-coated with a thin layer of gold for 30 s. The SEM was operated under the following conditions: accelerating voltage of 3 kV and working current of 5 mA.

### 2.8 Surface roughness measurement

Surface roughness of the films was characterized using a 3D optical surface profiler (ContourGT-K, Bruker). Film samples with a thickness of approximately 0.3 mm were used for testing. Measurements were performed at three different locations on the film surface. At each location, five scans were collected over a scan length of 0.8 mm and averaged. The reported roughness values represent typical measurements obtained under identical testing conditions.

### 2.9 X-ray diffraction (XRD)

The crystalline structure of the composites was analyzed by X-ray diffraction (XRD) using a D8-Advance diffractometer (Bruker, Germany). The measurements were performed with Cu Kα radiation (λ = 1.5418 Å), operating at 40 kV and 100 mA. The samples were scanned over a 2θ range from 5° to 50° at a scanning rate of 5°/min. The interplanar spacing ddd was calculated using Bragg's law:

(2-1) 2dsinθ=nλ

where

*d* = interplanar spacing,

*θ* = diffraction angle,

*λ* = wavelength of the X-rays, and

*n* = diffraction order.

From the measured diffraction angles, the lattice parameters and crystalline structure of the samples were determined.

### 2.10 Thermogravimetric analysis (TGA)

The thermal stability of the composites was assessed using a thermogravimetric analyzer (STA 409PC, NETZSCH, Germany). Film specimens (∼8–10 mg) were cut from molded sheets and placed in alumina pans. Heating program: 30 → 800 °C at 10 °C min^−1^ under N₂ (50 mL min^−1^). TG and DTG were recorded simultaneously. T₅% and T₁₀% were taken from TG; DTG peak maxima (T_max, stage-specific) were used as characteristic degradation temperatures.

### 2.11 Water absorption and moisture content

The water absorption (WU) and moisture content (ω) of the LDPE/CS composites were measured following standard procedures. Circular film specimens were prepared using a manual tablet press. The initial mass of the samples (ma) was recorded. Subsequently, the samples were dried in an oven at 105 °C for 4 h followed by drying at 85 °C for an additional 8 h, and the dried mass (mb) was measured. Each measurement was performed at least five times, and the results are reported as mean ± standard deviation. For the water absorption test, the dried samples were immersed in deionized water at room temperature for 24 h. After immersion, the samples were removed, surface water was gently wiped off with tissue paper, and the wet mass (mc) was recorded.

Water absorption (WU, %) was calculated using the following equation:

(2-2) $$\mathrm{WU}\left(\%\right)=\frac{m_{c}-m_{b}\;}{m_{b}\;}\times 100\%$$

Moisture content (ω, %) was calculated as:

(2-3) $$\omega \left(\%\right)=\frac{m_{a}-m_{b}\;}{m_{a}}\times 100\%$$

### 2.12 Water vapor and oxygen transmission rates

The water vapor barrier properties of the LDPE, M-LDPE, LDPE/CS and M-LDPE/CS composites were evaluated using a water vapor transmission rate (WVTR) testing system (model W3/060, Jinan Languang Electromechanical Technology Co., Ltd., Jinan, China), following the ASTM E398–13 and ASTM E96 standards. Circular film samples with a diameter of 75 mm and a thickness of approximately 0.3 ± 0.02 mm were prepared for testing. Each measurement was conducted at least three times to ensure reproducibility. The measurements were conducted at a controlled temperature of 25 °C with a chamber humidity of 89%. A testing interval of 30 min was used to ensure stabilization before recording mass changes.

To compare samples with different thicknesses, the water vapor permeability (WVP) coefficient was calculated using the following equation:

(2-4) $$P_{v}=\frac{∆m\cdot d}{A\cdot t\cdot ∆P}$$

where Pv = water vapor permeability, g·cm/cm2·s·Pa;

∆m = mass increment within a certain time t, g;

t = time interval after the mass increment stabilization, s;

d = sample thickness, cm;

A = area of sample available for water vapor permeation, cm2;

∆P = water vapor pressure difference across the sample, Pa.

### 2.13 Meat preservation test: total viable count (TVC)

Fresh meat samples were obtained from a local market and cut into pieces of similar size. The samples were randomly divided into groups and wrapped with different films (LDPE, LDPE/CS, M-LDPE, and M-LDPE/CS), while unwrapped samples were used as the control. All samples were stored under refrigerated conditions (8 ± 1 °C) without active humidity control for up to 96 h. At predetermined time intervals (24, 48, and 96 h), samples were collected for microbial analysis. For total viable count (TVC) determination, the samples were homogenized in sterile saline, serially diluted, and plated on nutrient agar. After incubation at 37 °C for 24 h, colony-forming units (CFU) were counted and expressed as CFU/mL. Each measurement was performed in triplicate.

### 2.14 Vegetable preservation test

The preservation performance and rate of water loss (RWL) of the LDPE, M-LDPE, LDPE/CS and M-LDPE/CS composite films were evaluated using fresh Chinese cabbage purchased from a local market. Any surface moisture was gently removed with dry tissue paper, and the initial weight (W0W_0W0) of the vegetables was recorded. The cleaned vegetables were then wrapped with the fabricated composite films, while unwrapped vegetables served as the control group. All samples were stored under ambient conditions (25 ± 1 °C and 70 ± 5% relative humidity), without active environmental control unless otherwise specified for a period of four days. After storage, the vegetables were reweighed to obtain the final weight. The rate of water loss (RWL) was calculated using the following equation:

(2-5) $$\mathrm{RWL}=\left(W_{0}-W_{\mathrm{dry}}\right)/W_{0}$$

*W*_0_ = initial weight

*W*_*dry*_ = the weight at room conditions (26 °C and 60% RH) for four days.

### 2.15 Banana preservation test: weight loss, pH, and total soluble solids (TSS)

Bananas of similar maturity (commercial ripening stage 4–5; uniform size and free of visible defects) were randomly assigned to five packaging groups: Control (unwrapped), LDPE, LDPE/CS_10, M-LDPE, and M-LDPE/CS_10. Each fruit was gently wiped with dry tissue to remove surface moisture prior to weighing. Fruits were wrapped with the designated films and stored at room conditions (26 ± 1 °C; 60 ± 5% RH), with temperature and humidity monitored throughout the storage period for up to 14 days without additional handling.

Weight loss. Individual fruits (*n* = 3 per group) were weighed on day 0, day 7, and day 14 using an analytical balance (±0.01 g). Weight loss was calculated as in Eq. (2–5) using the initial weight (W₀) and the weight at the specified time point (W_t). Results are reported as mean ± SD.

pH. On each sampling day (0, 7, 14), ∼20 g of pulp from the equatorial region of each fruit was homogenized with 40 g of deionized water (1:2, *w*/w) for 60 s. The slurry was filtered (Whatman No. 1), and the filtrate pH was measured at 25 °C with a calibrated benchtop pH meter (two-point calibration at pH 7.00 and 4.01 at the start of each session). Each fruit was measured in triplicate and averaged; group data are reported as mean ± SD (*n* = 3 fruits).

Total soluble solids (TSS). The same filtrate was used to determine TSS (°Brix) with a handheld digital refractometer at 25 °C. The instrument was zeroed with deionized water before each series. Three readings per fruit were recorded and averaged; group data are reported as mean ± SD (n = 3 fruits).

Visual assessment. High-resolution images were taken on days 0, 7, and 14 under consistent lighting and distance. Macroscopic features (peel color change, surface wrinkling/collapse, and visible mold) were qualitatively noted to complement gravimetric and physicochemical data.

## 3 Results and discussion

### 3.1 Tensile properties of LDPE/CS and M-LDPE/CS composites

**Fig. 2:**
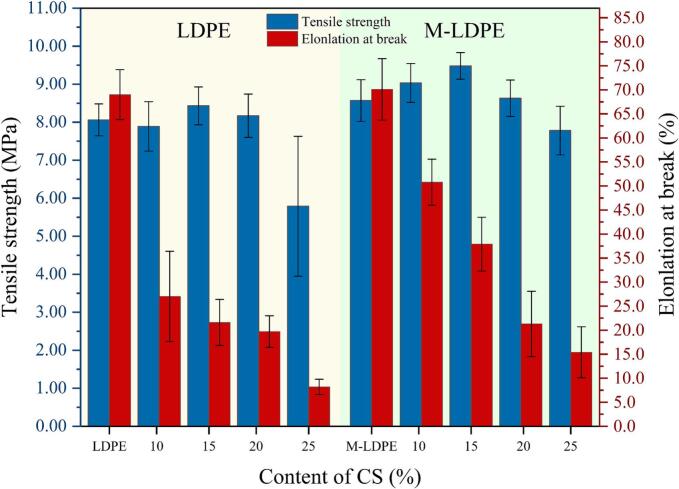
The tensile properties of LDPE/CS and M-LDPE/CS composites.

The tensile strength and elongation at break of LDPE/CS and M-LDPE/CS composites are illustrated in Fig. 2 and Table S1. The incorporation of cotton straw CS into LDPE resulted in a complex variation in tensile properties depending on the filler content.

For the LDPE/CS series, the addition of 10–20 wt% CS showed a slight increasing trend in tensile strength compared to neat LDPE (8.06 MPa), reaching a maximum of 8.43 MPa at 15 wt% CS. This trend is likely associated with the reinforcing effect of relatively well-dispersed CS fibers. However, when the CS content was further increased to 25 wt%, the tensile strength dropped significantly to 5.79 MPa, likely due to filler agglomeration and poor interfacial adhesion, which introduced stress concentration sites and weakened the composite structure (Ejeta et al., 2024; Tsou et al., 2024).

The elongation at break showed a sharp decline with increasing CS content. Neat LDPE exhibited an elongation of 69%, which dropped to 27% with 10 wt% CS and further decreased to 8.2% at 25 wt%. This trend indicates that the addition of rigid CS particles restricts the mobility of the polymer chains, resulting in reduced ductility (Lee et al., 2021; Shah et al., 2005).

In contrast, the M-LDPE/CS composites, which incorporated 4.5 wt% LDPE-g-MA as a compatibilizer, exhibited higher tensile strength overall and improved toughness compared to their non-compatibilized counterparts. The tensile strength peaked at 9.48 MPa for M-LDPE/CS_15, significantly higher than both neat LDPE and the corresponding LDPE/CS_15 sample. Even at 25 wt% CS, the tensile strength (7.78 MPa) remained much higher than that of LDPE/CS_25, demonstrating the efficacy of LDPE-g-MA in enhancing interfacial bonding between the CS fibers and the polymer matrix (Tsou, Chen, et al., 2022; Tsou, Ma, et al., 2022; Wang et al., 2019).

Similarly, the addition of LDPE-g-MA improved the elongation at break across all M-LDPE/CS samples. For example, at 10 wt% CS, M-LDPE/CS exhibited an elongation of 50.8%, compared to 27% in the unmodified LDPE/CS system. Although elongation decreased with higher CS content, M-LDPE/CS composites still maintained better ductility than their LDPE/CS equivalents, indicating that the compatibilizer effectively mitigated the embrittlement effect caused by filler addition. In summary, the results confirm that the use of LDPE-g-MA effectively improves the tensile strength and flexibility of LDPE/CS composites by enhancing interfacial compatibility and stress transfer between the matrix and the filler.

### 3.2 FTIR analysis

**Fig. 3:**
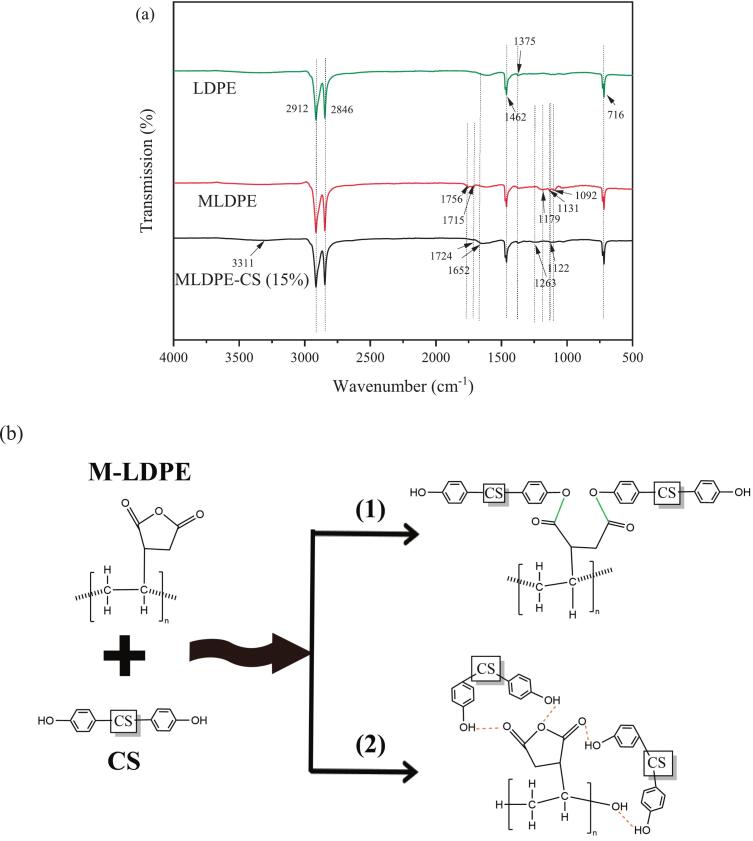
(a) FTIR spectra of LDPE, M-LDPE, and M-LDPE/CS composites; (b) schematic illustration of interfacial interactions between M-LDPE and CS.

The FTIR spectra of LDPE, M-LDPE, and M-LDPE/CS composites are presented in Fig. 3a. Neat LDPE shows its typical absorption peaks at 2912 and 2846 cm^−1^ (–CH₂ stretching), 1462 and 1375 cm^−1^ (–CH₂ bending), and 716 cm^−1^ (–(CH₂)ₙ– rocking) (Xu et al., n.d.; Chen et al., 2025; Umarie & Bagastyo, 2024). These bands confirm the structural fingerprint of LDPE.

After grafting with maleic anhydride, the spectrum of M-LDPE retains all major LDPE peaks, but new absorptions appear at 1756 and 1715 cm^−1^ (C=O stretching of anhydride and carboxyl groups) and at 1179, 1131, and 1092 cm^−1^ (C—O stretching of anhydride structures) (Liu et al., 2023; Scano, 2021). The emergence of these peaks indicates that maleic anhydride was introduced into the LDPE backbone.

When CS is incorporated into M-LDPE, significant spectral changes occur. The absorption band at 1756 cm^−1^ disappears completely, while the 1715 cm^−1^ peak shifts to 1724 cm^−1^ with reduced intensity. Moreover, a new peak emerges at 1652 cm^−1^, which can be assigned to C=O stretching of ester groups or conjugated carbonyls, suggesting possible interfacial interactions, likely involving reactions between the hydroxyl groups of CS and the anhydride groups of LDPE-g-MA. Meanwhile, the anhydride-related peaks at 1179, 1131, and 1092 cm^−1^ almost vanish, with new or shifted bands appearing at 1263 and 1122 cm^−1^. These changes are consistent with interfacial interactions and structural rearrangements at the filler–matrix interface, as commonly reported in compatibilized lignocellulose–polyolefin systems (Liu et al., 2023; Scano, 2021).

The proposed mechanism of interfacial interaction is illustrated in Fig. 3b. The grafted maleic anhydride groups of LDPE-g-MA are considered likely to interact with the abundant hydroxyl groups of CS, possibly forming covalent ester bonds, providing a strong chemical linkage between the hydrophobic polymer and the hydrophilic filler (Fig. 3b(1)). In addition, some hydroxyl groups on CS are able to form hydrogen bonds with residual anhydride or carboxyl groups, generating secondary interactions that further improve interfacial adhesion (Fig. 3b(2)). The coexistence of ester bonding and hydrogen bonding is believed to contribute to the enhanced compatibility, better dispersion, and more efficient stress transfer in M-LDPE/CS composites. This molecular-level interaction correlates well with the mechanical reinforcement observed in Section 3.1, particularly the higher tensile strength achieved at 15 wt% CS, where interfacial bonding and filler dispersion reached optimal balance. It should be noted that the FTIR results provide indirect evidence of these interactions, and the proposed mechanism is therefore presented as a plausible interpretation rather than direct chemical proof. It should be emphasized that ester bond formation in this system primarily improves interfacial adhesion and reduces interfacial defects, leading to a more compact structure. These structural changes influence diffusion pathways and, consequently, the transport behavior of gases and moisture, rather than involving any direct bactericidal or chemically active effect.

### 3.3 SEM morphology of LDPE, and LDPE/CS and M-LDPE/CS composites

**Fig. 4:**
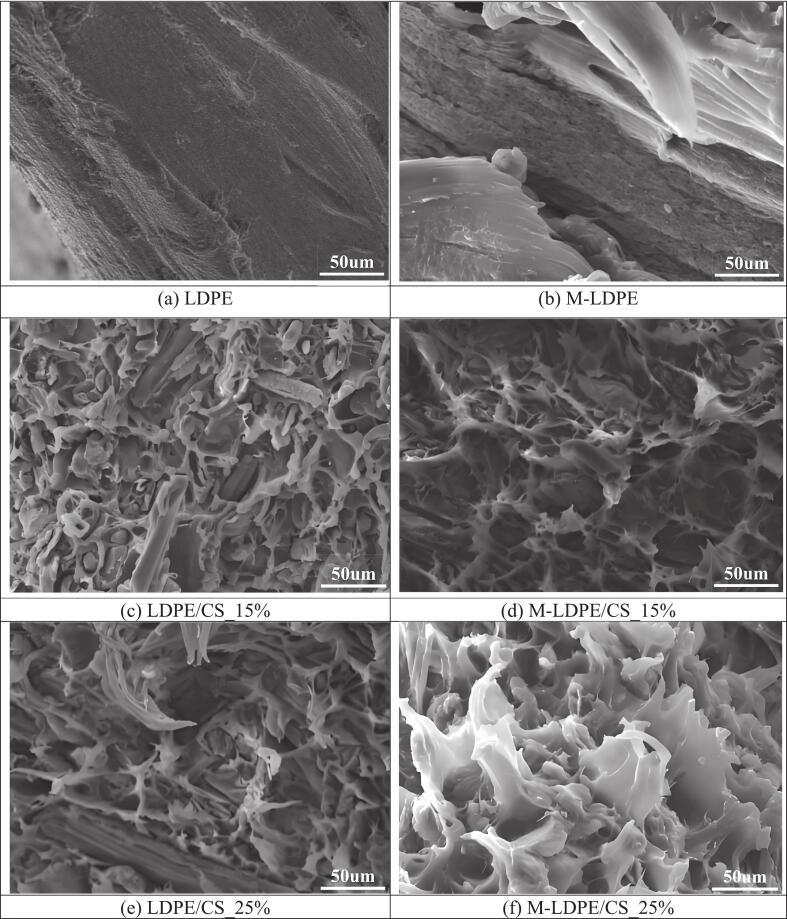
SEM images of LDPE, M-LDPE, LDPE/CS, and M-LDPE/CS composites, showing fracture surface morphologies for comparison.

The fracture cross-sectional morphologies of neat LDPE, M-LDPE, and their corresponding composites were examined by SEM, as shown in Fig. 4. Fig. 4a, c, and e present the LDPE-based systems (neat LDPE, LDPE/CS_15, and LDPE/CS_25), while Fig. 4b, d, and f correspond to the M-LDPE-based systems with the same compositions.

For neat LDPE (Fig. 4a), the fracture surface appears relatively smooth and continuous, indicating a typical ductile fracture behavior with limited resistance to crack propagation. After grafting with maleic anhydride, M-LDPE (Fig. 4b) shows a slightly rougher and more irregular morphology, suggesting that the introduction of polar functional groups may modify the deformation and fracture characteristics of the matrix, even in the absence of filler.

In the LDPE/CS composites (Fig. 4c and e), CS particles are embedded within the matrix, but clear interfacial gaps and pull-out features can be observed. These gaps become more pronounced at higher filler loading (25 wt%, Fig. 4e), where larger voids and discontinuities are evident. Such features indicate weak interfacial adhesion, arising from the intrinsic incompatibility between the hydrophilic CS and the hydrophobic LDPE. In addition, signs of filler aggregation can be observed at higher loading, which may introduce stress concentration points and negatively affect mechanical performance (Du et al., 2024; Khairkkar et al., 2025).

In contrast, the M-LDPE/CS composites (Fig. 4d and f) exhibit a more compact and cohesive fracture morphology. The interfaces between CS and the matrix appear tighter, with fewer observable voids and reduced pull-out features. This improvement is attributed to the interaction between the hydroxyl groups on CS and the anhydride groups in LDPE-g-MA, which can promote interfacial bonding and enhance compatibility (Shui et al., 2024; Tsou et al., 2024). As a result, the dispersion of CS is more uniform, and the continuity of the matrix is better preserved.

A notable feature in the M-LDPE/CS system, particularly at higher filler loading (Fig. 4f), is the presence of fibrillar or stretched structures within the matrix. These features, appearing as elongated and interconnected domains, are indicative of increased energy dissipation during fracture and suggest improved stress transfer at the filler–matrix interface (Varma & Chandran, 2025). The more developed fibrillar morphology in M-LDPE/CS_25 further supports the role of LDPE-g-MA in strengthening interfacial interactions and mitigating the negative effects of filler aggregation (Mohammed et al., 2022).

Overall, the SEM observations demonstrate that while the incorporation of CS alone leads to interfacial defects and reduced structural integrity at higher loadings, the use of LDPE-g-MA effectively improves interfacial bonding and dispersion. This structural evolution is consistent with the mechanical performance trends discussed in Section 3.1, confirming the importance of interfacial compatibilization in achieving balanced properties in LDPE-based composites. This structural difference is also reflected at the surface level. In particular, the improved interfacial bonding and reduced void formation in the M-LDPE/CS system are expected to influence surface morphology and roughness, which are further examined in the following section.

### 3.4 Surface roughness analysis

**Fig. 5:**
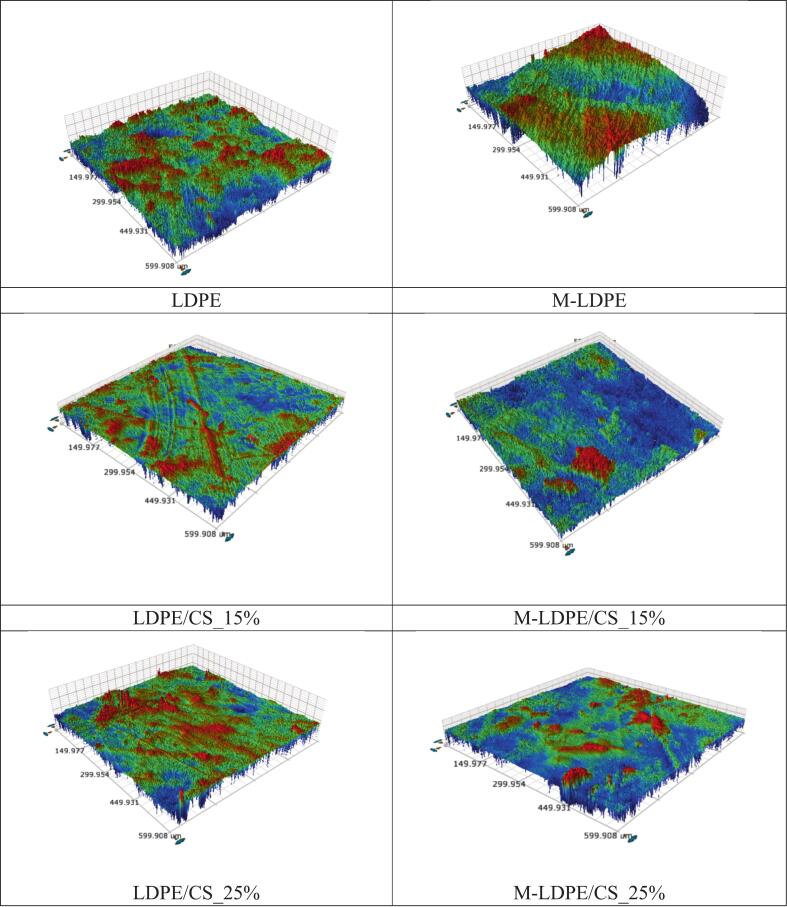
Surface roughness maps of LDPE, M-LDPE, LDPE/CS, and M-LDPE/CS films.

The surface roughness of LDPE, M-LDPE, and their corresponding composites was further characterized, and the results are shown in Fig. 5, with quantitative parameters summarized in Table S2.

Neat LDPE shows a relatively high surface roughness (Sa ≈ 1368 nm), which is consistent with its heterogeneous surface features. After grafting with maleic anhydride, M-LDPE exhibits a lower roughness (Sa ≈ 999 nm), suggesting a more uniform surface compared to LDPE. After introducing CS, the roughness of LDPE/CS composites decreases (Sa ≈ 422–481 nm), indicating that the filler may partially smooth the surface, possibly by filling surface irregularities. In contrast, the M-LDPE/CS system shows a different trend. At 15 wt% CS, the roughness increases (Sa ≈ 2073 nm), while at higher loading (25 wt%), the roughness decreases again (Sa ≈ 870 nm). This change may be related to filler aggregation or structural rearrangement at higher loading levels.

Overall, the roughness results are consistent with the SEM observations. The LDPE/CS system tends to show a smoother surface, while the M-LDPE/CS system exhibits more pronounced surface variations. These differences are associated with interfacial compatibilization and may influence moisture interaction and mass transport behavior, as discussed in the following sections.

### 3.5 XRD analysis and crystallinity of LDPE, M-LDPE, and M-LDPE/CS composites

**Fig. 6:**
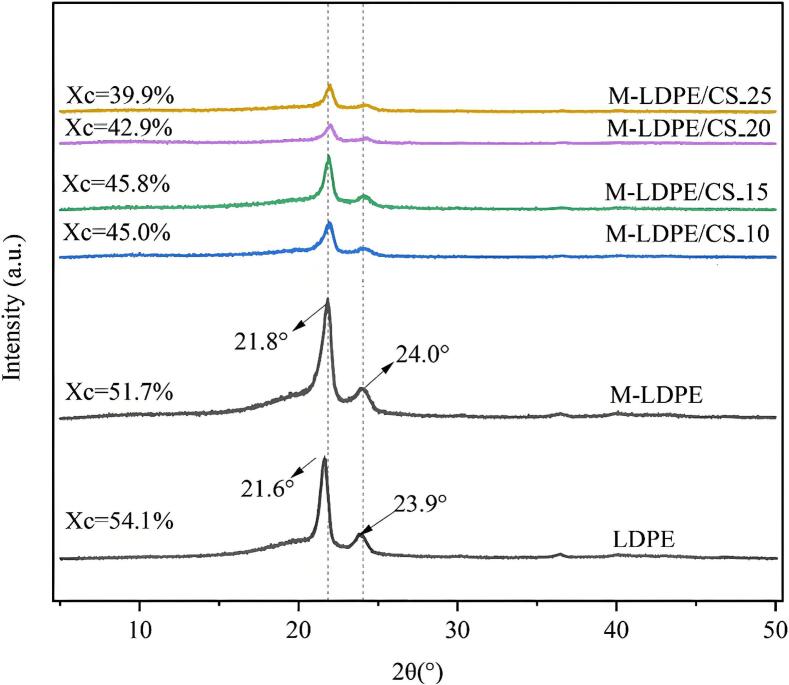
XRD of LDPE-CS composites.

Fig. 6 presents the XRD patterns of neat LDPE, M-LDPE, and M-LDPE/CS composites with varying CS content. All samples exhibit two distinct diffraction peaks in the range of 21–24°, corresponding to the (1 1 0) and (2 0 0) crystallographic planes of LDPE, confirming the semi-crystalline nature of the polymer matrix (Depan et al., 2021). For neat LDPE, the diffraction peaks are located at 21.6° and 23.9°, with a crystallinity of 54.1%, representing the baseline crystalline order of the unmodified polymer.

Upon grafting with maleic anhydride (M-LDPE), the characteristic diffraction peaks shift slightly to 21.8° and 24.0°, accompanied by a marginal reduction in peak intensity. The corresponding crystallinity decreases to 51.7%, indicating that the grafted MA units disturb chain packing and slightly lower crystalline order compared to neat LDPE.

With the incorporation of CS, further changes in peak intensity and sharpness were observed. At 10 wt% CS, M-LDPE/CS_10 exhibited a reduced crystallinity of 45.0%. Interestingly, at 15 wt% CS, crystallinity reached 45.8% and the diffraction peaks became more pronounced, indicating localized improvement in ordering. However, further increases in CS content to 20 and 25 wt% led to significant peak broadening, with crystallinity decreasing to 42.9% and 39.9%, respectively, consistent with increased amorphous fractions and disrupted crystal packing.

The evolution of crystallinity closely parallels the tensile strength trend discussed in Section 3.1. M-LDPE/CS_15 exhibited the highest crystallinity among the filled composites (45.8%) and simultaneously achieved the maximum tensile strength (9.48 MPa). This suggests that an optimal filler content of 15 wt% enables a balance between lamellar ordering and interfacial reinforcement (Albedah et al., 2024; Griffin et al., 2022). At low loading (10 wt%), the filler effect is insufficient to form an effective reinforcing network, while at higher loadings (≥20 wt%), excessive CS induces heterogeneity, agglomeration, and restricted chain mobility, leading to diminished crystallinity and mechanical properties (Abdul Khalil et al., 2019; Taguet et al., 2014).

In summary, neat LDPE exhibits peaks at 21.6° and 23.9° with a crystallinity of 54.1%. After grafting and filler incorporation, M-LDPE/CS composites maintain the two main crystalline peaks at ∼21.8° and ∼24.0°, but the crystallinity gradually decreases with increasing CS content, peaking at 15 wt% as the most favorable balance between structural regularity and interfacial adhesion.

### 3.6 TGA analysis

**Fig. 7:**
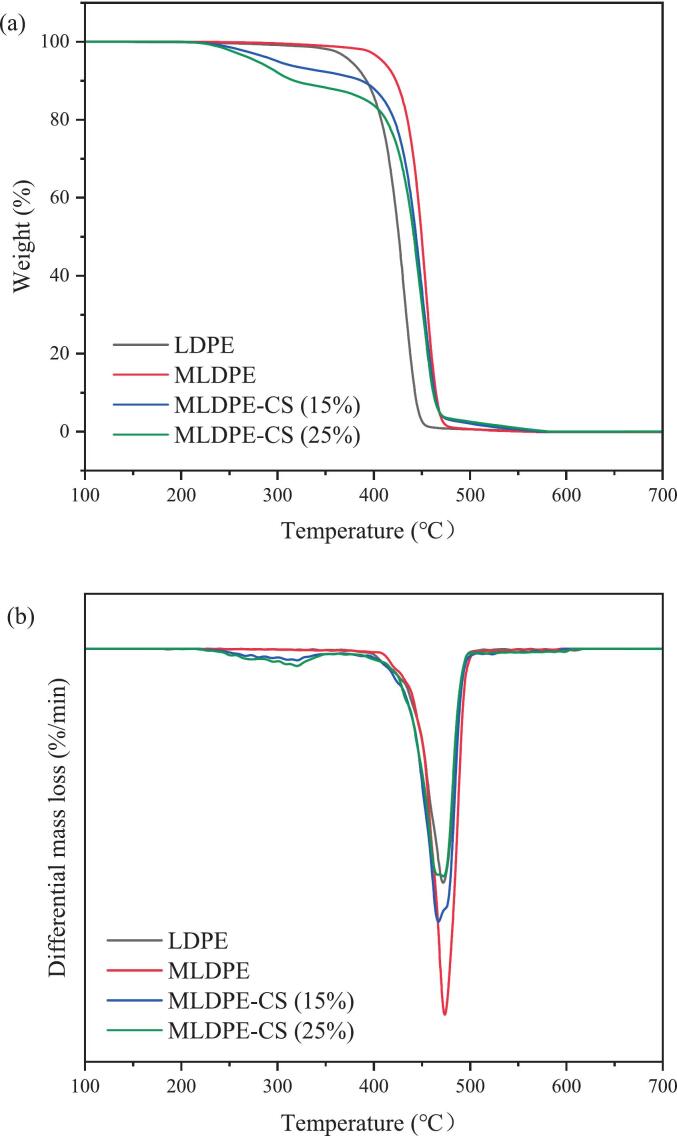
(a) TG and (b) DTG curves of LDPE, M-LDPE, and M-LDPE/CS composites.

The thermal stability of LDPE, M-LDPE, and M-LDPE/CS composites was evaluated by TGA and DTG (Fig. 7a and b), and the key parameters are summarized in Table 2. Neat LDPE exhibits a single major degradation stage with a T_5_% of 375.8 °C and a T_10_% of 392.3 °C. The maximum degradation rate occurs at 472.8 °C (Tmax_2), which is characteristic of polyethylene chain scission (Hernandez-Hosaka et al., 2025). After grafting with maleic anhydride, M-LDPE shows slightly improved thermal stability, with T₅% and T₁₀% shifted to 408.9 °C and 423.0 °C, respectively, while the main degradation peak (Tmax_2) remains close to that of LDPE (474.1 °C). This enhancement is attributed to the presence of polar MA groups, which increase chain interactions and restrict thermal motion to some extent (Murillo & López, 2015; Shang et al., 2025).

**Table 2:** TGA of LDPE, M-LDPE and M-LDPE/CS composites.

Samples	T5% (°C)	T10% (°C)	Tmax_1 (°C)	Tmax_2 (°C)
LDPE	375.8	392.3	/	472.82
MLDPE	408.9	423.0	/	474.11
MLDPE-CS (15%)	299.4	386.9	321.2	460.47
MLDPE-CS (25%)	278.5	315.8	320.6	472.80

In contrast, incorporation of CS into M-LDPE significantly alters the degradation behavior. For M-LDPE/CS composites, two distinct DTG peaks are observed: a minor shoulder peak (Tmax_1) at ∼321 °C and a stronger main peak (Tmax_2) between 460 and 473 °C. The first peak corresponds to the decomposition of hemicellulose, cellulose, and partially lignin components from CS, while the second peak reflects the degradation of the LDPE matrix. The presence of this bimodal degradation pattern confirms the combined contributions of both natural filler and polymer to the thermal response.

At moderate loading (15 wt% CS), M-LDPE/CS_15 shows a T₅% of 299.4 °C and a T₁₀% of 386.9 °C, indicating earlier onset of weight loss compared with neat M-LDPE. However, this initial weight loss is primarily associated with the thermal decomposition of lignocellulosic components in CS and occurs at temperatures far exceeding those encountered during food packaging processing or service conditions. Nevertheless, the main degradation peak (Tmax_2) shifts slightly lower to 460.5 °C, reflecting a partial compromise of matrix stability due to filler incorporation. At higher loading (25 wt% CS), the reduction in initial stability is more pronounced, with T₅% and T₁₀% dropping to 278.5 °C and 315.8 °C, respectively. Interestingly, the main peak (Tmax_2) recovers to 472.8 °C, nearly identical to neat LDPE, while the first cellulose-related peak (Tmax_1) remains around 320.6 °C (Arnandan et al., 2025; Garcia-Vargas et al., 2025). This suggests that although CS accelerates initial degradation by introducing thermally less stable components, the overall char residue formation and filler–matrix interactions can help stabilize the polymer phase during the later stages of decomposition. Importantly, this two-step degradation behavior does not compromise the thermal safety or functional integrity of the films under typical food packaging and storage conditions, which are well below the onset temperatures observed here.

In summary, the TGA results reveal a trade-off between filler incorporation and thermal stability. LDPE-g-MA alone improves the onset stability of LDPE, while the addition of CS lowers the early degradation temperature but introduces a characteristic two-step decomposition process. The balance between matrix reinforcement and filler-induced instability is especially evident at 15–25 wt% CS, where the composites display reduced onset temperatures yet retain relatively high Tmax₂ values. These findings highlight that interfacial compatibility and filler loading are critical factors in determining the overall thermal performance of M-LDPE/CS composites, while also confirming that the observed reduction in initial degradation temperature does not pose a practical limitation for food packaging applications, given the substantial margin between degradation onset and real processing or service temperatures.

### 3.7 Contact angle of LDPE/CS composites and M-LDPE/CS composites

**Fig. 8:**
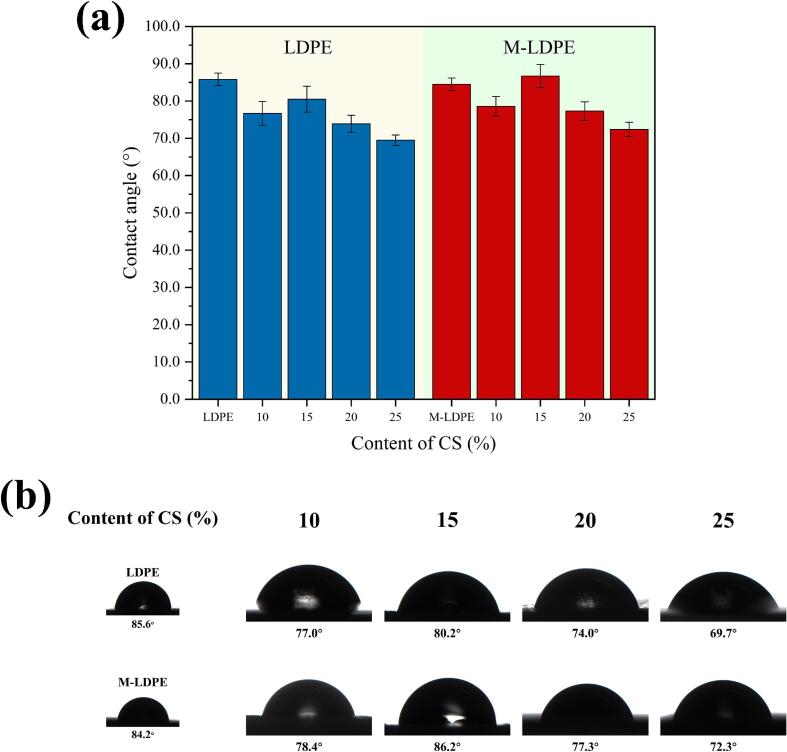
The Contact angle of LDPE/CS Composites and M-LDPE/CS composites.

The water contact angle measurements of LDPE/CS and M-LDPE/CS composites are and illustrated in Fig. 8. These measurements provide insights into the surface wettability and hydrophilic/hydrophobic balance of the composites.

In the LDPE/CS composites, the incorporation of hydrophilic cotton straw (CS) fibers leads to a general decrease in contact angle compared to neat LDPE (85.8°). For instance, LDPE/CS_10 exhibits a contact angle of 76.7°, and this value further decreases to 69.5° for LDPE/CS_25. This trend indicates that the addition of CS enhances the surface hydrophilicity of the composites. The hydrophilic nature of plant fibers like CS is attributed to their abundant hydroxyl groups, which have a high affinity for water molecules (Atmakuri et al., 2020).

Conversely, the M-LDPE/CS composites, which incorporate LDPE-g-MA as a compatibilizer, display a different behavior. Notably, M-LDPE/CS_15 exhibits a contact angle of 86.7°, surpassing that of neat LDPE. This increase suggests that the compatibilizer enhances interfacial adhesion between the CS fibers and the LDPE matrix, leading to a more cohesive and less porous surface (Lee et al., 2021). Improved interfacial bonding reduces the exposure of hydrophilic fiber ends on the composite surface, thereby increasing the overall hydrophobicity (Ma et al., 2022).

Additionally, surface roughness plays a significant role in determining the wettability of polymer composites. According to the Wenzel model, increased surface roughness can amplify the inherent wettability of a material. In the case of LDPE/CS composites, the presence of CS fibers may introduce surface irregularities, contributing to the observed decrease in contact angle (Mohammed et al., 2022; Wenzel, 1949). However, in M-LDPE/CS composites, the enhanced compatibility between the matrix and the fibers results in a smoother surface morphology, mitigating the effects of roughness on wettability.

In summary, the incorporation of CS fibers into LDPE matrices increases the surface hydrophilicity of the composites, as evidenced by decreased contact angles. However, the use of LDPE-g-MA as a compatibilizer in M-LDPE/CS composites counteracts this effect by improving interfacial adhesion and reducing surface roughness, leading to contact angles comparable to that of neat LDPE. It should be noted that water contact angle reflects surface wettability at the air–film interface, whereas water content and water absorption characterize bulk moisture uptake governed by internal interfacial structure and diffusion pathways, as discussed in the following section.

### 3.8 Water content and water absorption of LDPE/CS of LDPE/CS and M-LDPE/CS composites

**Fig. 9:**
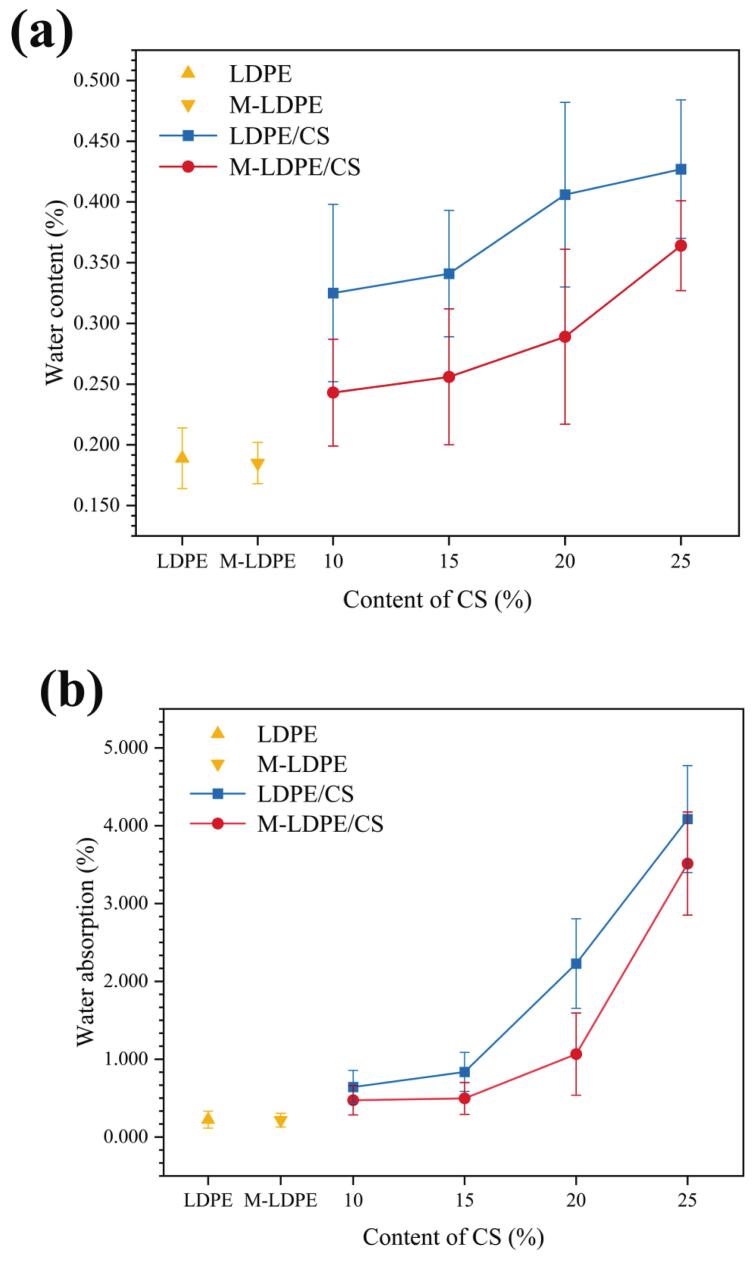
(a) Water content and (b) water absorption of LDPE/CS and M-LDPE/CS composites.

The water content and water absorption behaviors of LDPE/CS and M-LDPE/CS composites are depicted in Fig. 9a and b, respectively. These two parameters reflect moisture uptake under ambient conditions and immersion, respectively. In the LDPE/CS composites, both water content and water absorption increase consistently with higher CS loading. For instance, water content increased from 0.189% in pure LDPE to 0.427% in LDPE/CS_25, while water absorption rose sharply from 0.223% to 4.085%. This trend is mainly attributed to the hydrophilic nature of CS, which contains abundant hydroxyl groups that attract water. In addition, filler-induced microvoids may facilitate moisture diffusion (Karthik et al., 2024; Wang, Chen, et al., 2023).

In contrast, M-LDPE/CS composites showed significantly lower moisture sensitivity at comparable CS contents. At 25 wt% CS, the water content and absorption of M-LDPE/CS composites were 0.364% and 3.514%, respectively—both lower than those of the uncompatibilized LDPE/CS composites. This improvement is mainly due to the incorporation of LDPE-g-MA, which enhances interfacial adhesion between the hydrophilic CS fibers and the hydrophobic LDPE matrix. Stronger interfacial bonding reduces the number of interfacial voids and hinders moisture penetration pathways (Sahu & Gupta, 2022; Thirumalaisamy et al., 2023).

The effect of compatibilization is especially evident at moderate CS loadings (10–20 wt%), where M-LDPE/CS composites maintain relatively low water absorption (<1.1%), in contrast to the steep increase observed in LDPE/CS counterparts. This suggests that LDPE-g-MA not only promotes better dispersion of the filler but also contributes to a more compact and moisture-resistant composite structure. In summary, CS increases moisture uptake, while LDPE-g-MA mitigates this effect by improving interfacial adhesion and reducing diffusion pathways.

### 3.9 Water vapor permeability of LDPE/CS and M-LDPE/CS composites

**Fig. 10:**
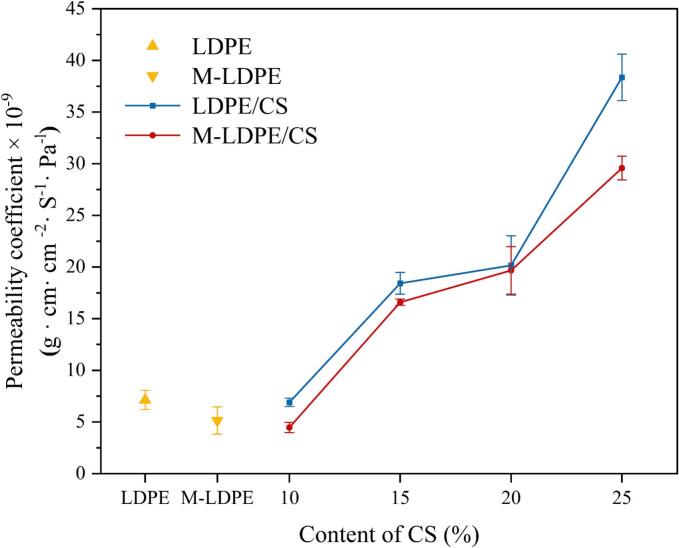
The water vapor permeability coefficient of LDPE/CS and M-LDPE/CS composites.

The water vapor permeability (WVP) coefficients of LDPE/CS and M-LDPE/CS composites are illustrated in Fig. 10. These values reflect the material's barrier performance against moisture transmission, which is critical for food packaging and other moisture-sensitive applications.

In the LDPE/CS composites, a clear trend emerges: as the content of cotton straw (CS) increases, the WVP coefficient rises noticeably. Pure LDPE exhibited a relatively low permeability of 7.14 × 10^−9^ g^3^·cm·cm^−2^·s^−1^·Pa^−1^. The addition of 10 wt% CS caused a slight reduction in permeability (6.90 × 10^−9^), likely due to partial blockage of diffusion pathways by dispersed filler. However, when the CS content exceeded 15 wt%, permeability increased clearly, reaching 18.43 × 10^−9^ at 15 wt%, 20.16 × 10^−9^ at 20 wt%, and peaking at 38.36 × 10^−9^ for LDPE/CS_25. This sharp increase is likely associated with the hydrophilicity of CS, interfacial defects at higher loadings, and possible filler aggregation, all of which facilitate vapor diffusion (Candra et al., 2023; Du et al., 2025; Lang et al., 2024; Mohammed et al., 2023; Qin et al., 2022).

In contrast, M-LDPE/CS composites exhibit clearly improved water vapor barrier properties, especially at low to moderate CS loadings. The WVP of pure M-LDPE is only 5.14 × 10^−9^, already lower than that of neat LDPE, and further drops to 4.47 × 10^−9^ for M-LDPE/CS_10. This improvement is likely associated with LDPE-g-MA, which enhances interfacial compatibility and contributes to the reduction of effective diffusion pathways (Arman Alim et al., 2023; Feng et al., 2019).

However, as the CS content increases beyond 15 wt%, WVP also begins to rise in M-LDPE/CS samples, albeit to a lesser extent than in the uncompatibilized system. At 25 wt% CS, M-LDPE/CS still maintains a lower WVP value (29.58 × 10^−9^) compared to LDPE/CS_25 (38.36 × 10^−9^). This indicates that although high CS loading compromises the barrier property due to intrinsic hydrophilicity and structural disruption, the presence of the compatibilizer can still partially mitigate the adverse effects.

In summary, the incorporation of CS increases the water vapor permeability of LDPE-based composites, particularly at high filler content. However, LDPE-g-MA effectively improves dispersion and reduces interfacial defects, particularly at low to moderate loadings.

### 3.10 Oxygen transmission rate (OTR)

**Fig. 11:**
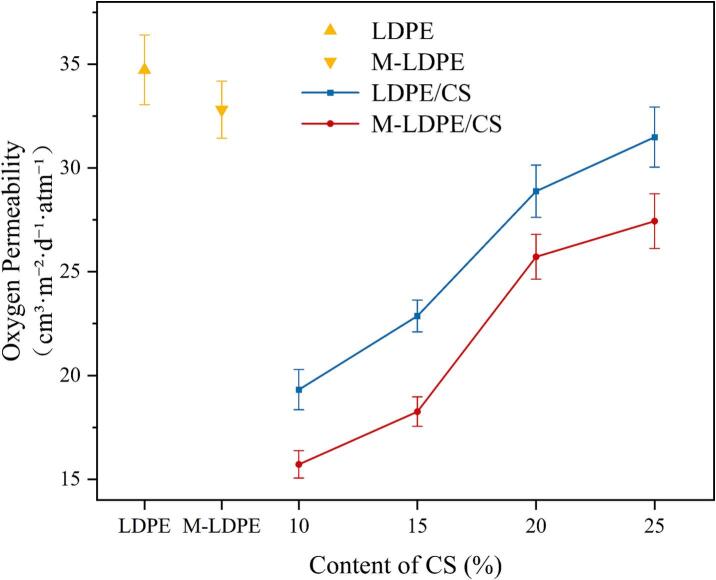
The oxygen permeability of LDPE/CS and M-LDPE/CS composites.

Rather than pursuing maximal barrier performance, this system maintains moderate moisture permeability to avoid dehydration stress. To assess small-molecule gas barrier performance, oxygen permeability was measured for all films (Fig. 11). In both LDPE/CS and M-LDPE/CS series, adding cotton straw (CS) lowers OTR, with the largest drop at 10–15 wt% CS; at higher loadings (≥20 wt%), OTR rises slightly but remains below the neat matrix.

Relative to neat LDPE (34.73 cm^3^·m^−2^·d^−1^·atm^−1^), LDPE/CS_10 decreases to 19.32 (≈44% reduction) and LDPE/CS_15 to 22.87. Further increases to 20–25 wt% bring OTR up to 28.88 and 31.49, still below LDPE. For the compatibilized system, M-LDPE shows a baseline OTR of 32.81; introducing 10 wt% CS yields the lowest value in this work (15.72; ≈52% below M-LDPE and ≈55% below LDPE), while 15 wt% gives 18.26. At 20–25 wt%, OTR increases to 25.72–27.44, yet remains well below the non-compatibilized counterparts (e.g., ≈13% lower than LDPE/CS_25).

This ‘decrease–then–slight rebound’ profile suggests a tortuosity-related transport behavior.

Here, “tortuosity” is used in a qualitative sense to describe the extension of diffusion pathways, rather than a quantitatively determined parameter. At 10–15 wt% CS, finely dispersed particles extend diffusion pathways and modestly tune crystalline order (consistent with the 15 wt% optimum in XRD), producing OTR minima (Kwon et al., 2024). At ≥20 wt%, local aggregation and interfacial heterogeneity erode those gains (Tan & Thomas, 2017). Unlike water vapor, oxygen permeability appears to be mainly influenced by diffusion tortuosity and interfacial compactness; thus, interfacial densification and path tortuosity outweigh hygroscopic effects. LDPE-g-MA enhances both by promoting ester-bonding at the interface, suppressing voids, and stabilizing filler dispersion—hence the consistently lower OTR in M-LDPE/CS at matched CS loadings, in line with SEM evidence of tighter interfaces and fibrillation under load. These interpretations are based on the observed permeability trends and structural features, rather than a quantitative transport model.

Overall, the most favorable oxygen barrier window is 10–15 wt% CS, with “compatibilization + moderate loading” emerging as the practical recipe for coupling barrier performance with structural integrity. Thus, M-LDPE/CS at 10–15 wt% represents the most practical balance between barrier performance and mechanical integrity.

### 3.11 Schematic illustration of barrier mechanisms for water vapor and oxygen

**Fig. 12:**
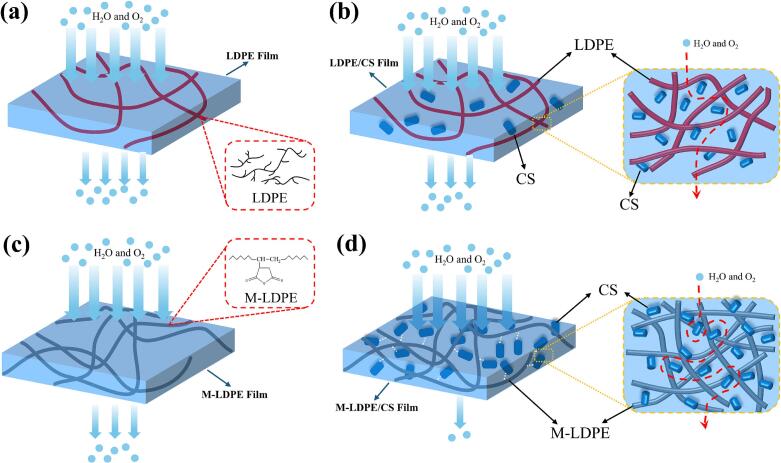
Schematic illustration of the proposed transport mechanisms of water vapor and oxygen in (a) LDPE, (b) LDPE/CS, (c) M-LDPE, and (d) M-LDPE/CS composite films. Incorporation of CS introduces tortuous diffusion pathways that predominantly hinder oxygen transport, while its hydrophilic nature influences water vapor transmission through moisture adsorption and localized diffusion. The presence of LDPE-g-MA promotes interfacial densification via chemical bonding with CS, reducing interfacial voids and free volume. Consequently, oxygen permeability appears to be mainly governed by diffusion tortuosity and interfacial compactness, whereas water vapor permeability reflects a balance between tortuosity and moisture–polymer interactions, resulting in optimal barrier performance at moderate CS loadings. These interpretations are based on the observed permeability trends and structural features, rather than a quantitative transport model.

Based on the permeability results presented in Sections 3.6 (WVP) and 3.7 (OTR), a schematic model was developed to illustrate the transmission pathways of water vapor and oxygen in representative films (Fig. 12).

In neat LDPE (Fig. 12a), water vapor and oxygen diffuse through relatively direct pathways, resulting in moderate permeability (Qu et al., 2023). With the incorporation of 10 wt% CS (LDPE/CS_10), dispersed fillers partially obstruct and elongate the diffusion paths (Fig. 12b); however, limited interfacial adhesion introduces micro voids that restrict the overall barrier enhancement (Ge et al., 2025; Wang, He, et al., 2023).

For M-LDPE (Fig. 12c), the introduction of maleic anhydride groups enhances matrix cohesion and reduces free volume, slightly lowering WVP and OTR compared to neat LDPE (Sarbazi et al., 2024). When CS is combined with LDPE-g-MA (M-LDPE/CS_10), a combined effect is observed (Fig. 12d). Ester bond formation between hydroxyl groups of CS and anhydride groups of LDPE-g-MA likely reduces interfacial defects, producing a denser and more compact structure. The improved interfacial structure forces both molecules to follow more tortuous paths, leading to lower permeability.

It should be noted that water vapor transmission is strongly affected by the hydrophilic chemistry of CS. In contrast, oxygen transport in LDPE/CS composites appears to be mainly governed by diffusion tortuosity and interfacial compactness, while water vapor transport is additionally influenced by hygroscopic domains in the lignocellulosic filler. At higher loadings (≥20 wt%), excessive hydroxyl groups and structural heterogeneity increase moisture uptake and create new penetration channels, leading to higher WVP despite increased tortuosity (Candra et al., 2023; Lang et al., 2024; Mohammed et al., 2023; Qin et al., 2022). Oxygen, being nonpolar, is less sensitive to hydrophilic sites; its transport is governed primarily by tortuosity and interfacial compactness. This is consistent with the observed decrease in OTR at 10–15 wt% CS but rises again at higher loadings due to filler agglomeration and microvoid formation.

In summary, the schematic highlights two main mechanisms for barrier enhancement: (i) the tortuous diffusion pathway induced by well-dispersed CS fibers, and (ii) interfacial densification facilitated by LDPE-g-MA compatibilization. These effects are maximized at moderate CS contents (10–15 wt%), providing the best balance between structure and barrier performance. The observed differences in barrier performance are consistent with the structural evolution revealed by SEM and surface roughness analysis. The more compact interfacial structure and modified surface morphology in the M-LDPE/CS system are likely to influence diffusion pathways and moisture interaction, contributing to the coupled regulation of gas and moisture transport.

### 3.12 Microbial response of LDPE/CS and M-LDPE/CS composites

Although cotton straw (CS) has not been widely reported as an intrinsic antimicrobial agent, the effect of LDPE/CS and M-LDPE/CS composite films on bacterial viability was evaluated against *Escherichia coli* (*E. coli*) to examine whether CS incorporation and interfacial modification could influence bacterial survival at the film surface. Bacterial viability was assessed by enumerating colony-forming units (CFU/mL) after incubation.

**Fig. 13:**
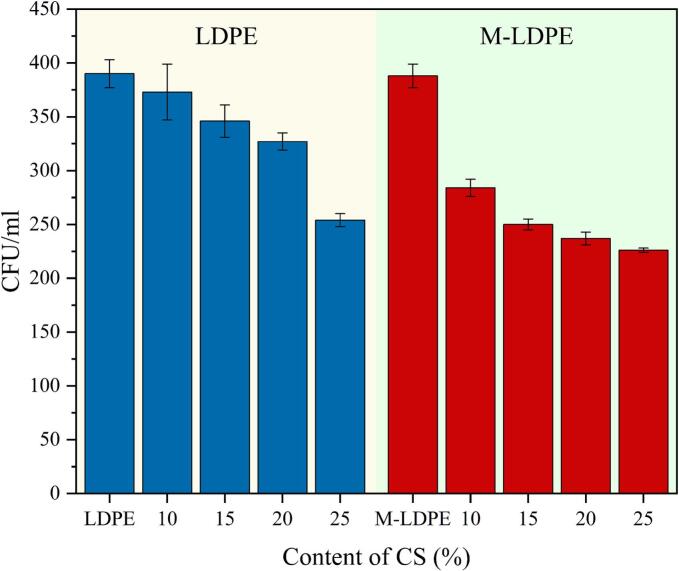
Viable *E. coli* colony counts (CFU/mL) on LDPE, LDPE/CS, and M-LDPE/CS composite films after incubation, showing gradual suppression of bacterial proliferation with increasing CS content and enhanced effects in compatibilized systems.

As shown in Fig. 13, neat LDPE exhibited a CFU count of approximately 390 CFU/mL, indicating no obvious effect on bacterial viability. With increasing CS content, a gradual reduction in viable bacterial colonies was observed. For example, LDPE/CS_10 showed a modest decrease to 373 CFU/mL, while LDPE/CS_25 reached 254 CFU/mL. This trend suggests that CS does not act as an antimicrobial component but may influence bacterial survival indirectly through changes in surface wettability, microstructure, and local moisture conditions, rather than through direct antibacterial action.

Compared with the uncompatibilized systems, M-LDPE/CS composites exhibited a more pronounced reduction in bacterial viability. Notably, M-LDPE/CS_25 showed the lowest CFU count (226 CFU/mL). This improvement is likely associated with the role of LDPE-g-MA in promoting interfacial densification and surface uniformity through chemical bonding with CS, rather than being related to any direct antimicrobial effect. Improved interfacial compatibility reduces microvoids and limits favorable niches for bacterial attachment and growth.

Previous studies have occasionally reported that maleic anhydride functionalities may interact with microorganisms under certain conditions; however, these effects are generally weak and are not regarded as a direct antimicrobial mechanism (Tsou et al., 2019; Tsou et al., 2023). However, in the present system, LDPE-g-MA is incorporated as a compatibilizer within the polymer backbone, and its contribution to the reduction in bacterial growth is considered indirect. The observed reduction in bacterial growth is therefore associated with the combined effects of interfacial densification, modified surface chemistry, and moisture-regulating characteristics, which collectively create a less favorable environment for microbial proliferation.

In summary, CS incorporation into LDPE films contributes to a reduction in bacterial growth, which becomes more evident when combined with LDPE-g-MA compatibilization. Rather than functioning as an active antimicrobial material, the M-LDPE/CS system influences bacterial proliferation through structural and microenvironmental effects. This restrained behavior is advantageous for food-packaging applications, as it enhances preservation performance without relying on metallic or migratory antimicrobial additives. This behavior is consistent with the structural and transport characteristics discussed above, where interfacial densification, surface morphology, and mass transport collectively contribute to a less favorable microenvironment for microbial proliferation.

### 3.13 Total viable count analysis of meat samples wrapped with LDPE/CS and M-LDPE/CS films

**Fig. 14:**
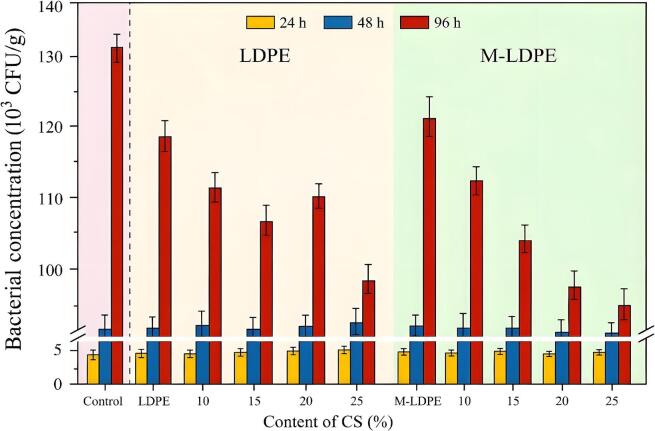
Total viable counts of meat samples stored under refrigeration for 24, 48, and 96 h using different composite films. M-LDPE/CS composites showed the greatest reduction in microbial growth compared to LDPE and LDPE/CS films. Data are presented as mean ± standard deviation (*n* = 3).

Fig. 14 presents the results of the total viable count (TVC) of meat samples stored under identical refrigerated conditions (8 °C) for 24, 48, and 96 h using different packaging films. The control group (no film) showed a sharp increase in microbial load over time, reaching 1.33 × 10^5^ CFU/mL at 96 h. In contrast, all film-wrapped samples exhibited lower microbial growth compared to the control, indicating a measurable preservation effect.

LDPE-based films moderately delayed microbial proliferation compared to the control. LDPE/CS composites further reduced bacterial counts across all timepoints, with LDPE/CS_25 reaching only 1.00 × 10^5^ CFU/mL at 96 h (vs. 1.21 × 10^5^ CFU/mL for neat LDPE). M-LDPE/CS composites showed the greatest reduction, with M-LDPE/CS_25 achieving 9.9 × 10^4^ CFU/mL at 96 h, suggesting a combined contribution of the compatibilizer and the CS filler. The observed preservation behavior is likely associated with several factors, rather than a single dominant mechanism.

(1) Intrinsic and Chemical Factors: CS, as a lignocellulosic material containing polyphenolic and waxy components, may have a minor influence on microbial growth, although its direct antimicrobial effect is limited. LDPE-g-MA may also contribute slightly through its polar functional groups, which can interact with microbial membranes. Together, these effects are likely to play a minor but non-negligible role in the reduced microbial growth observed in M-LDPE/CS films.
(2) Barrier and Moisture Regulation Effects: The reduction in microbial proliferation is consistent with the trends observed in water vapor permeability. Films with improved barrier properties (e.g., M-LDPE/CS_10 and M-LDPE/CS_15) help limit moisture exchange, reducing humid conditions favorable for bacterial growth. At the same time, the hydrophilic nature of CS can adsorb moisture on the film surface, lowering free water availability at the meat–film interface and indirectly suppressing microbial propagation (Du et al., 2024).
(3) Structural Compactness and Nutrient Availability: The strong interfacial bonding promoted by LDPE-g-MA leads to denser, less porous composite structures, which can hinder oxygen and moisture diffusion — critical factors for microbial survival. Additionally, this compact interface may limit the release of nutrients or leachates from the film matrix, further restricting microbial proliferation on the surface.

In summary, the combination of CS and LDPE-g-MA in these packaging films is associated with delayed microbial growth and improved preservation performance under chilled conditions. Nevertheless, further studies are recommended to explore a broader range of pathogens and commercial storage scenarios to validate these promising results.

### 3.14 Visual appearance and weight loss of vegetables wrapped with LDPE/CS and M-LDPE/CS composite films

**Fig. 15:**
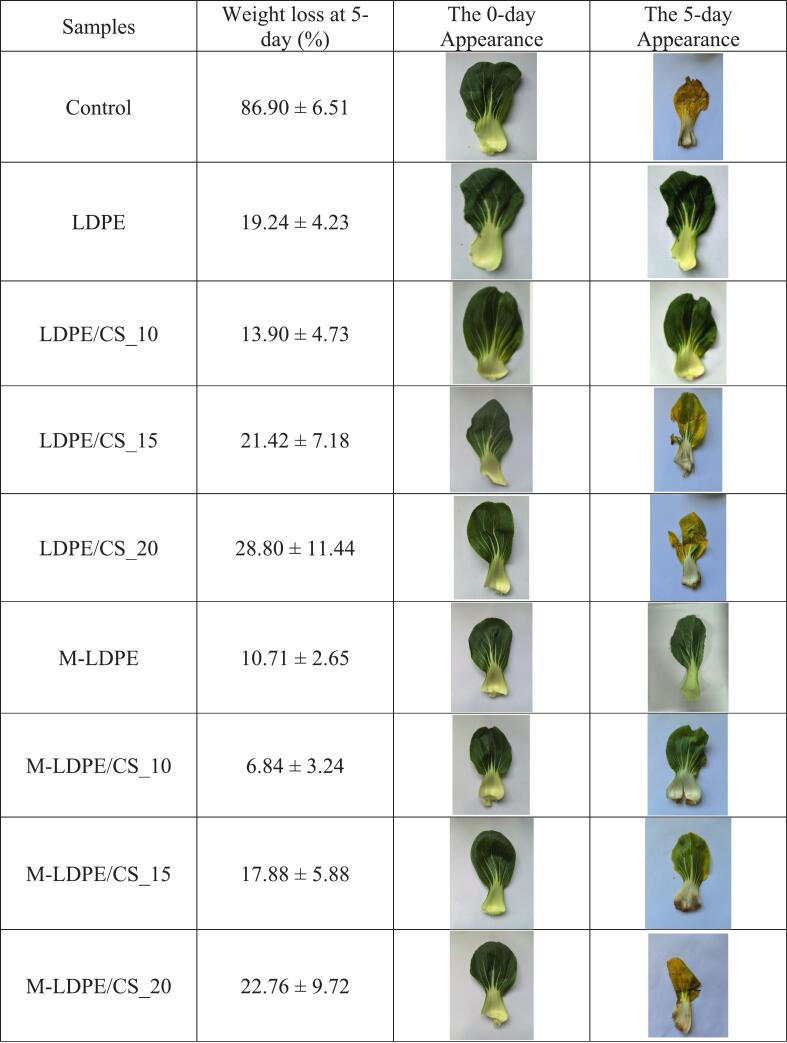
Visual appearance and weight loss of vegetables wrapped with LDPE, LDPE/CS, M-LDPE, and M-LDPE/CS composite films after 5 days of storage. Data are presented as mean ± standard deviation (*n* = 3). Different letters indicate significant differences (*p* < 0.05).

To evaluate the preservation performance of the developed packaging films, fresh vegetables were wrapped and stored under ambient conditions—approximately 26 °C and 60% relative humidity—for 5 days. The weight loss was measured as an indicator of moisture retention capability, and visual inspection was conducted to assess dehydration and freshness (Fig. 15).

The control sample (unwrapped) showed severe moisture loss, with a weight reduction of 86.9%, confirming rapid dehydration in the absence of packaging (Pascall, 2010). Vegetables wrapped with neat LDPE had a significantly lower weight loss of 19.24%, while the addition of 10 wt% CS further reduced the loss to 13.9%, likely due to the moisture adsorption and partial barrier function provided by CS.

However, as CS content increased beyond 10 wt%, the LDPE/CS_15 (21.42%) and LDPE/CS_20 (28.8%) films became less effective. This is possibly due to the increase in hydrophilic sites and interfacial voids caused by excessive filler, which could promote water vapor diffusion instead of inhibiting it.

M-LDPE-based films performed better overall. Neat M-LDPE reduced weight loss to 10.71%, and M-LDPE/CS_10 further reduced it to 6.84%, the lowest among all samples. This suggests improved water vapor barrier performance due to both improved interfacial compatibility and optimal filler content. However, further increasing the CS content (to 15–20 wt%) diminished the benefit, with weight losses rising to 17.88% and 22.76%, respectively.

Visual differences also clearly support the weight loss data. The control vegetable appeared flattened, wrinkled, and yellowed, indicating extreme water loss and tissue degradation. LDPE/CS_15, LDPE/CS_20, and M-LDPE/CS_20 showed moderate surface dryness and visible yellowing, suggesting that excessive filler loading weakened their barrier integrity. In contrast, vegetables wrapped with LDPE/CS_10, M-LDPE, and especially M-LDPE/CS_10 retained firmness, color, and freshness, with minimal visible water loss. These results suggest that M-LDPE/CS_10 has strong potential for short-term, room-temperature food packaging applications requiring high moisture retention and visual preservation.

### 3.15 Visual appearance, weight loss, and physicochemical changes of bananas wrapped with LDPE/CS and M-LDPE/CS composite films

**Fig. 16:**
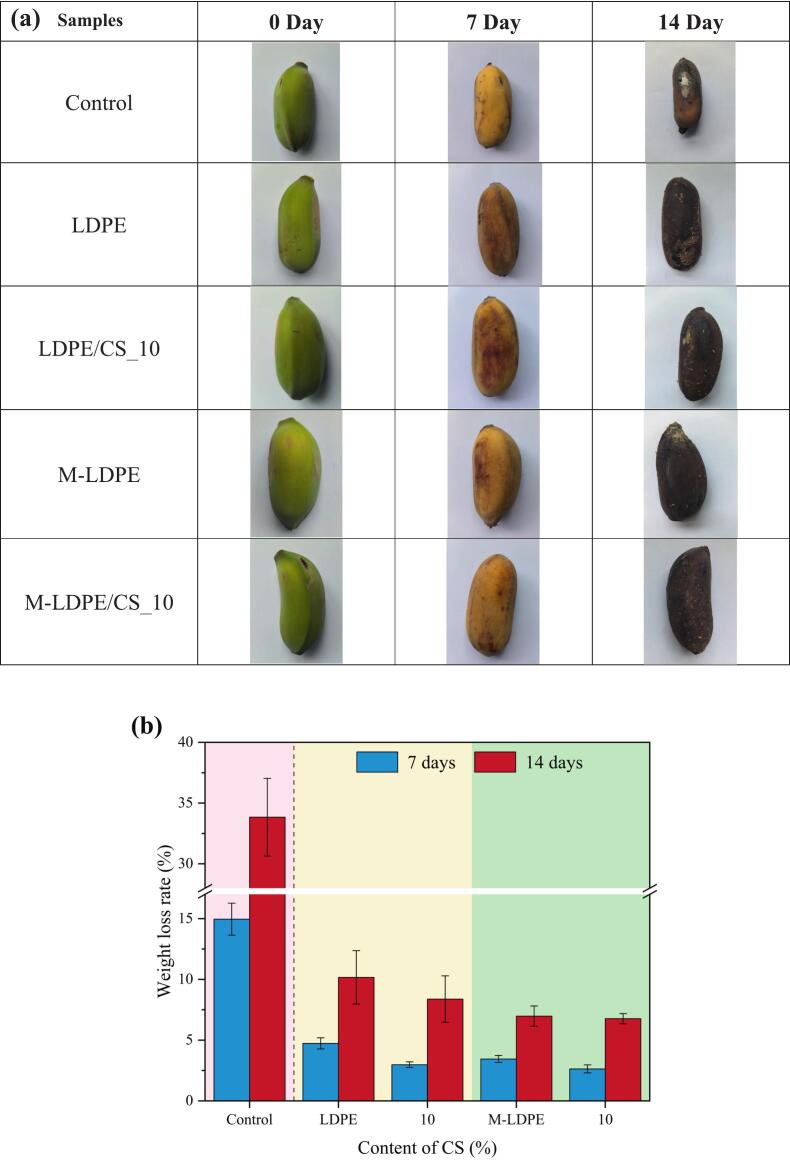
(a) Visual appearance and (b) weight loss of bananas wrapped with LDPE, LDPE/CS, M-LDPE, and M-LDPE/CS composite films after 7 and 14 days of storage. All images are presented at the same scale.

To evaluate the suitability of LDPE/CS and M-LDPE/CS composite films for fruit packaging, bananas were wrapped with different films and stored at room temperature (∼26 °C, 60% RH) for up to 14 days. Weight loss was measured on days 7 and 14 as an indicator of moisture retention, while visual appearance was assessed to monitor freshness, ripening, and spoilage. Fig. 16 shows (a) the visual appearance and (b) the weight loss of bananas after 7 and 14 days of storage.

As shown in Fig. 16b, bananas in the control group (unwrapped) exhibited the most severe moisture loss, with 14.96% weight loss at day 7, increasing to 33.84% at day 14. This rapid water loss resulted in visible shrinkage, tissue collapse, and accelerated spoilage. All wrapped samples displayed significantly lower weight loss. LDPE-wrapped fruits showed 4.73% and 10.17% loss, respectively. Incorporation of CS into LDPE further reduced weight loss; for example, LDPE/CS_10 exhibited 2.98% at day 7 and 8.38% at day 14. M-LDPE and M-LDPE/CS composites showed even better retention. Notably, M-LDPE/CS_10 achieved the lowest values, with only 2.63% at day 7 and 6.77% at day 14.

Although weight loss generally decreased with higher CS content, the differences among CS-containing groups were relatively small, suggesting that beyond a certain level, additional CS has limited effect on water retention. However, across both film systems, M-LDPE/CS consistently outperformed uncompatibilized counterparts, confirming the role of LDPE-g-MA in enhancing barrier integrity.

At day 0, all bananas appeared green and unripe, with no visible differences. By day 7, most fruits had turned yellow, indicating normal ripening, and no obvious differences were observed among the wrapped groups. By day 14, all bananas had become dark brown or black, reflecting overripening. Nevertheless, distinct differences in structural integrity and microbial contamination were observed:

(1) The control banana showed severe shrinkage, dryness, and visible white mold, indicating microbial spoilage and advanced dehydration due to lack of packaging (Hailu et al., 2014). (2) In contrast, all wrapped bananas (both LDPE- and M-LDPE-based) maintained structural integrity and remained free of visible mold even after 14 days. Particularly, M-LDPE/CS_10 retained better shape, with less surface darkening and wrinkling (Rodov et al., 2022; Sharma et al., 2024).

In addition to weight loss and visual appearance, total soluble solids (TSS) and pulp pH were monitored to further evaluate the physicochemical stability of bananas during storage (Table S3 and Table S4). In the control group, TSS increased sharply from 3.7°Brix to 17.8°Brix after 14 days (*p* < 0.001), reflecting rapid starch hydrolysis and sugar accumulation during overripening. LDPE packaging moderated this change, with final TSS reduced to 12.3°Brix (p < 0.001). Incorporation of CS further suppressed sugar accumulation, as shown by LDPE/CS_10 with 11.1°Brix at day 14 (p < 0.001). The strongest effect was observed for compatibilized films: M-LDPE and M-LDPE/CS_10 limited TSS increases to 10.7°Brix and 8.0°Brix, respectively (both p < 0.001), confirming their ability to delay ripening-related biochemical conversion.

The pH results followed a similar trend. The control group showed a pronounced increase from 3.12 to 5.34 over 14 days, consistent with metabolic shifts during senescence. LDPE and LDPE/CS_10 slowed this progression, with final pH values of 5.14 and 5.21, but these increases were not statistically significant compared with the control (*p* = 0.12 and *p* = 0.32, respectively). By contrast, M-LDPE and M-LDPE/CS_10 significantly suppressed the rise in pulp pH, reaching only 4.92 (*p* < 0.01) and 4.51 (p < 0.01) after 14 days. These results indicate that compatibilized composites more effectively maintained the biochemical stability of the fruit by reducing the extent of acid degradation and overripening.

Taken together, the TSS and pH analyses reinforce the weight-loss and appearance data, showing that M-LDPE/CS_10 films not only minimized moisture loss but also slowed sugar accumulation and restrained pH increases, thereby extending freshness and delaying spoilage more effectively than uncompatibilized films.

## 4 Conclusion

In this study, cotton straw (CS) was successfully utilized as a multifunctional bio-filler in LDPE-based packaging films through a process compatible with conventional polyolefin manufacturing. The introduction of LDPE-g-MA improved interfacial compatibility, resulting in enhanced dispersion, interfacial densification, and modified surface morphology. Rather than introducing a new tortuosity mechanism, this work demonstrates a coupled regulation framework, in which interfacial structure and surface characteristics jointly influence gas transport, moisture behavior, and the microbial microenvironment. SEM and surface roughness analyses consistently reveal that compatibilization leads to a more integrated structure and distinct surface features, which are closely associated with the observed transport and preservation behavior.

At 10–15 wt% CS, the composites exhibited an optimal balance of mechanical properties, oxygen barrier performance, and moisture regulation. This translated into improved food preservation performance, including reduced weight loss, stabilized quality parameters, and delayed microbial growth. The preservation effect is not attributed to direct antimicrobial activity, but rather to structural densification and microenvironment regulation. From an application perspective, the proposed system offers a scalable and practical approach for incorporating agricultural residues into LDPE-based packaging. Cotton straw provides a low-cost and abundant raw material, while the processing route remains compatible with existing polyolefin manufacturing without requiring complex modifications. Although the addition of LDPE-g-MA introduces a slight increase in material cost, its low loading and the resulting performance enhancement justify its use.

Overall, this work highlights the importance of integrating interfacial design and surface structure regulation to achieve multi-property optimization in bio-filled packaging systems, providing a feasible pathway toward sustainable and functional polyolefin-based food packaging.

## CRediT authorship contribution statement

**Chi-Hui Tsou:** Writing – review & editing, Supervision, Methodology, Investigation, Funding acquisition, Conceptualization. **Nuo Xu:** Writing – original draft, Project administration, Methodology, Investigation, Data curation. **Jia Zheng:** Visualization, Resources, Investigation, Formal analysis. **Genjun Ye:** Validation, Resources, Investigation. **Lin-Kai Wu:** Validation, Software, Data curation. **Xin Huang:** Investigation, Formal analysis. **Tao Guo:** Visualization, Resources. **Yulong Luo:** Resources, Investigation. **Guangfu Mao:** Resources, Project administration. **Xue-Fei Hu:** Resources, Project administration, Investigation, Funding acquisition.

## Funding

This work was supported by the Cooperation Project between Wuliangye Group Co., Ltd. and Sichuan University of Science & Engineering, China (CXY2022ZR001); the Scientific Research and Innovation Team Program of Sichuan University of Science & Engineering; the 10.13039/100012542Sichuan Province Science and Technology Support Program (2022JDTD0016); and the Open Foundation of the Key Laboratory of Wuliangye-Flavor Liquor Solid-State Fermentation, China National Light Industry (2024JJ004). Supported by The Innovation Fund of Postgraduate, Sichuan University of Science & Engineering.

## Declaration of competing interest

The authors declare that they have no known competing financial interests or personal relationships that could have appeared to influence the work reported in this paper.

## Data Availability

The data that support the findings of this study are available from the corresponding author upon reasonable request.
