# Supplementary material for: Toward sustainable LDPE packaging films from cotton straw: barrier–moisture–microenvironment coupling for food preservation
Source: Food Chem X. 2026 Apr 24;36:103917. doi: 10.1016/j.fochx.2026.103917 (PMC13185940; doi:10.1016/j.fochx.2026.103917)
Supplement: Supplementary file 1 — Supplementary material. [file mmc1.docx]

Supplementary materials

Toward Sustainable LDPE Packaging Films from Cotton Straw: Barrier–Moisture–Microenvironment Coupling for Food Preservation

Chi-Hui Tsou^1*+^，Nuo Xu^1+^， Jia Zheng ^2^， Genjun Ye^2^，Lin-Kai Wu^1^,

Xin Huang^1^，Tao Guo^2^， Yulong Luo^2^，Guangfu Mao^2^， Xue-Fei Hu^1*^

^1^ Material Corrosion and Protection Key Laboratory of Sichuan Province, School of Materials Science and Engineering, Sichuan University of Science and Engineering, Zigong 643000, China

^2^ Wuliangye Yibin Co., Ltd. Yibin, Sichuan 644000, China

* Correspondence: mayko0301@hotmail.com (Chi-Hui Tsou);

+ These authors contributed equally to this work.

| Samples | Tensile strength (MPa) | Elongation at break (%) |
| --- | --- | --- |
| LDPE | 8.06 ± 0.42 | 69 ± 5.2 |
| LDPE/CS_10 | 7.89 ± 0.65 | 27 ± 9.4 |
| LDPE/CS_15 | 8.43 ± 0.50 | 21.6 ± 4.8 |
| LDPE/CS_20 | 8.17 ± 0.57 | 19.7 ± 3.3 |
| LDPE/CS_25 | 5.79 ± 1.84 | 8.2 ± 1.6 |
| M-LDPE | 8.57 ± 0.55 | 70.1 ± 6.4 |
| M-LDPE/CS_10 | 9.03 ± 0.51 | 50.8 ± 4.8 |
| M-LDPE/CS_15 | 9.48 ± 0.35 | 37.9 ± 5.6 |
| M-LDPE/CS_20 | 8.63 ± 0.48 | 21.3 ± 6.8 |
| M-LDPE/CS_25 | 7.78 ± 0.64 | 15.4 ± 5.3 |

Table S1. The Tensile properties of LDPE/CS and M-LDPE/CS composites (Values represent mean ± standard deviation (n ≥ 5).

Table S2. Surface roughness parameters of LDPE, M-LDPE, LDPE/CS, and M-LDPE/CS films. Values represent typical measurements obtained under identical testing conditions. Sa: arithmetic mean height; Sq: root mean square height; Sp: maximum peak height; Sv: maximum valley depth; Sz: maximum height difference; Ssk: skewness; Sku: kurtosis.

| Samples | Labe | | | | | | |
| --- | --- | --- | --- | --- | --- | --- | --- |
|  | Sa (nm) | Sku | Sp (nm) | Sq (nm) | Ssk | Sv (nm) | Sz (nm) |
| LDPE | 1368.655 | 4.265 | 15684.339 | 1793.58 | 0.368 | -9446.23 | 25130.568 |
| LDPE/CS_15 | 422.474 | 4.368 | 5197.861 | 538.937 | 0.438 | -11347.268 | 16545.129 |
| LDPE/CS_25 | 481.387 | 12.253 | 10370.13 | 650.767 | 1.052 | -8277.922 | 18648.054 |
| M-LDPE | 999.536 | 2.808 | 4991.678 | 1207.795 | 0.171 | -7772.819 | 12764.498 |
| M-LDPE/CS_15 | 2073.487 | 6.961 | 16002.591 | 2886.085 | 1.58 | -10709.854 | 26712.446 |
| M-LDPE/CS_25 | 870.429 | 11.971 | 11332.332 | 1207.508 | 1.533 | -17507.484 | 28839.816 |

Table S3. Effect of LDPE, LDPE/CS, M-LDPE and M-LDPE/CS_10 composite films on the Total soluble solids (TSS) of banana over a storage period of 14 days at ambient condition. Statistical significance was determined by one-way ANOVA followed by Tukey’s post hoc test, with comparisons made between each packaged sample and the unwrapped control at the same storage time.

| Sample | Total soluble solids (°Brix) | | |
| --- | --- | --- | --- |
|  | 0 day | 7 days | 14 days |
| Control | 3.7 ± 0.2 | 11.3 ± 0.5 (p<0.01) | 17.8 ± 0.7 (p<0.001) |
| LDPE | 3.6 ± 0.2 | 9.2 ± 0.4 (p<0.001) | 12.3 ± 0.5 (p<0.001) |
| LDPE/CS_10 | 3.5 ± 0.3 | 8.7 ± 0.2 (p<0.001) | 11.1 ± 0.3 (p<0.001) |
| M-LDPE | 3.6 ± 0.2 | 8.6± 0.3 (p<0.001) | 10.7 ± 0.3 (p<0.001) |
| M-LDPE/CS_10 | 3.7 ± 0.1 | 5.4 ± 0.5 (p<0.001) | 8.0 ± 0.4 (p<0.001) |

Table S4. Effect of LDPE, LDPE/CS, M-LDPE and M-LDPE/CS_10 composite films on the pulp pH of banana over a storage period of 14 days at ambient condition. Statistical significance was determined by one-way ANOVA followed by Tukey’s post hoc test, with comparisons made between each packaged sample and the unwrapped control at the same storage time.

| Sample | pH | | |
| --- | --- | --- | --- |
|  | 0 day | 7 days | 14 days |
| Control | 3.12 ± 0.02 | 4.13 ± 0.03 | 5.34 ± 0.21 |
| LDPE | 3.18 ± 0.02 | 3.94 ± 0.04 (p<0.01) | 5.14± 0.12 (p=0.12, ns) |
| LDPE/CS_10 | 3.08 ± 0.03 | 3.86 ± 0.05 (p<0.01) | 5.21 ± 0.16 (p=0.32, ns) |
| M-LDPE | 3.05 ± 0.03 | 3.81 ± 0.04 (p<0.001) | 4.92 ± 0.13 (p<0.01) |
| M-LDPE/CS_10 | 3.11 ± 0.02 | 3.63 ± 0.03 (p<0.001) | 4.51 ± 0.08 (p<0.01) |

ns: not statistically significant (p > 0.05).
